# The Therapeutics of Glycozone, Composition and Characteristics

**Published:** 1894-02

**Authors:** Cyrus Edson

**Affiliations:** Health Commissioner, Board of Health, New York City


### Selections and Abstracts

### THE THERAPEUTICS OF GLYCOZONE, COMPOSITION AND CHARACTERISTICS.

By CYRUS EDSON, M. D.

Health Commissioner, Board of Health, New York City.

(Published by the *Times and Register*, Philadelphia, Pa., April 22, 1893.)

Glycozone is defined by its discoverer, Mr. Ch. Marchand, to be a stable compound, resulting from the chemical reaction that takes place when c. p. glycerine is submitted, under certain conditions, to the action of fifteen times its own volume of ozone, under normal atmospheric pressure at a temperature of 0°C.

The necessity of using c. p. glycerine is imperative, as the presence of water or other foreign matter in the glycerine causes the production in the resulting compound of formic acid, glyceric acid, and other secondary products, that have a harmful effect upon animal tissues.

Glycozone has a pleasant, sweetish taste. Being hygroscopic it must be kept in tightly corked bottles, and, as long as it is kept in this condition, it does not deteriorate at a temperature of even 110 degrees F.

*Antagonists and Incompatibles.*—Glycozone, like peroxide of hydrogen is a powerful oxidizing agent, although its action is not as rapid or as energetic in this respect as the latter compound. Consequently, we cannot safely prescribe it combined with any other drugs or chemical substances. Contact with metallic utensils decompose it. We must therefore use glass or hard rubber vessels and syringes when administering it.

*Physiological Action.*—When taken into the mouth and stomach glycozone causes a feeling of warmth. It excites a flow of saliva and stimulates the gastric secretions. Being hygroscopic it attracts to itself water from the surrounding tissues but not with sufficient power to effect harm. This property is due solely to the glycerine base which enters into the composition. In very large doses, one or two ounces, it causes a feeling of distress in the epigastrium and is followed by loose, copious, watery stools, which are accompanied by severe cramps.

No effect is noted on the kidneys, the liver or the heart. Glycozone is undoubtedly slowly decomposed in the stomach, ozone being liberated and the glycerine uniting with the water from the tissues. The morbid elements with which it comes in contact probably hasten this decomposition, and in so doing are themselves oxidized and destroyed. The free ozone in the stomach resulting from the decomposition of glycozone aids the digestive process by its presence.

*Therapy.*—Glycozone is, in the opinion of the writer, the best known agent for the treatment of gastric ulcer. It is also one of the best remedies for the treatment of the stomach, catarrh of chronic alcoholism, and for chronic gastric catarrh from other causes. It is excellent for atonic dyspepsia, and for acid dyspepsia. The writer has seen very gratifying results from its use in these distressing maladies.

In catarrhal and other stomachic diseases except gastric ulcer, the remedy is best administered in one or two teaspoonfuls in a wineglassful of water immediately after meals. In the case of gastric ulcer the dose and dilution should be the same, but it is better to give it when the stomach is empty, an hour or so before meals.

Glycozone has an excellent effect when used internally in cases of diphtheria. For this purpose a tablespoonful of glycozone is given in a wineglassful of water every three hours. As it is perfectly harmless it may be used without apprehension. The following treatment is excellent in cases of membranous croup: The nose, throat, mouth, pharynx, and larynx should be sprayed copiously every two hours or so with a mixture of one ounce of Marchand's peroxide of hydrogen (medicinal), with four to six ounces of water.

The membranes are readily destroyed, and by using this remedy freely, their reproduction is prevented. Then one teaspoonful of glycozone, diluted in a wineglassful of water, administered three times a day, prevents any disturbance of the stomach and regulates the bowels.

Remarkable benefit may be derived in the treatment of diseased conditions ( ulceration and chronic inflammation ) of the rectum and lower gut, by enemata containing glycozone, and for this purpose nothing excels the following formula :

Glycozone, 1 ounce.
Water, lukewarm, 12 ounces.

This should be mixed immediately before using and administered with a hard rubber syringe once daily. It is frequently desirable to use a smaller amount than the above mixture. The proportions one to twelve, however, should be maintained. In cases of fistula in ano and of rectal ulcerations low down, an ounce of lukewarm water containing a drachm of glycozone administered once or twice daily soon effects good and in cases of ulcer, pure and simple, may be expected to radically cure the diseased conditions.

*External Uses.*—After the cleansing of any diseased or suppurating surface by peroxide of hydrogen (medicinal), the application of glycozone stimulates healthy action and hastens the cure. For this purpose it has no superior in the entire range of therapeutics. It tends to check the discharge of irritating unwholesome secretions and to prevent the infection of the sore by pathogenic organisms. Its action in this respect is explained by the fact that it is both powerfully antiseptic and stimulant.

*Follicular Pharyngitis*, chronic coryza and ulcerative stomatitis are all benefited by frequent applications of glycozone. As an application to ulcerated cervix-uteri and in tumefied conditions of the cervix and uterus it is far superior to pure glycerine.

In these cases, and for the cure of leucorrhea, the remedy should be applied on small rolls of lint, or absorbent cotton, the vagina having first been thoroughly washed with an injection of peroxide of hydrogen one part, water four parts. This procedure should be repeated twice daily.

